# A knowledge management tool for public health: health-evidence.ca

**DOI:** 10.1186/1471-2458-10-496

**Published:** 2010-08-18

**Authors:** Maureen Dobbins, Kara DeCorby, Paula Robeson, Heather Husson, Daiva Tirilis, Lori Greco

**Affiliations:** 1 Faculty of Health Sciences, McMaster University, 1200 Main St. W., Hamilton, Ontario, Canada

## Abstract

**Background:**

The ultimate goal of knowledge translation and exchange (KTE) activities is to facilitate incorporation of research knowledge into program and policy development decision making. Evidence-informed decision making involves translation of the best available evidence from a systematically collected, appraised, and analyzed body of knowledge. Knowledge management (KM) is emerging as a key factor contributing to the realization of evidence-informed public health decision making. The goal of health-evidence.ca is to promote evidence-informed public health decision making through facilitation of decision maker access to, retrieval, and use of the best available synthesized research evidence evaluating the effectiveness of public health interventions.

**Methods:**

The systematic reviews that populate health evidence.ca are identified through an extensive search (1985-present) of 7 electronic databases: MEDLINE, EMBASE, CINAHL, PsycINFO, Sociological Abstracts, BIOSIS, and SportDiscus; handsearching of over 20 journals; and reference list searches of all relevant reviews. Reviews are assessed for relevance and quality by two independent reviewers. Commonly-used public health terms are used to assign key words to each review, and project staff members compose short summaries highlighting results and implications for policy and practice.

**Results:**

As of June 2010, there are 1913 reviews in the health-evidence.ca registry in 21 public health and health promotion topic areas. Of these, 78% have been assessed as being of strong or moderate methodological quality. Health-evidence.ca receives approximately 35,000 visits per year, 20,596 of which are unique visitors, representing approximately 100 visits per day. Just under half of all visitors return to the site, with the average user spending six minutes and visiting seven pages per visit. Public health nurses, program managers, health promotion workers, researchers, and program coordinators are among the largest groups of registered users, followed by librarians, dieticians, medical officers of health, and nutritionists. The majority of users (67%) access the website from direct traffic (e.g., have the health-evidence.ca webpage bookmarked, or type it directly into their browser).

**Conclusions:**

Consistent use of health-evidence.ca and particularly the searching for reviews that correspond with current public health priorities illustrates that health-evidence.ca may be playing an important role in achieving evidence-informed public health decision making.

## Background

The ultimate goal of knowledge translation and exchange (KTE) activities is to facilitate the incorporation of research knowledge into program and policy development decision making processes. The term implies that effective strategies are all that are needed to achieve this end. It is also understood that research knowledge, if only effectively translated, could inform policy and practice decisions and subsequently improve health outcomes [1]. In fact, more than ten years ago a key recommendation arising from the Canadian National Forum on Health [2] was the development of an evidence informed health care system in Canada where policies and clinical decisions are influenced by high quality research knowledge. Evidence-informed decision making involves the translation of the best available evidence from a systematically collected, appraised, and analyzed body of knowledge [3]. It is defined as a process characterized by: 1) clearly articulating a research question derived from a practice-based issue; 2) searching for and accessing relevant evidence; 3) appraising methodological rigor and choosing evidence of the highest quality with relevance to the practice issue and setting; 4) extracting evidence and interpreting it with consideration given to local context and resources; 5) incorporating it into practice, program, and policy decisions; and 6) evaluating the results [3,4]. Despite political will, significant challenges in the past decade have impeded efforts in the realization of evidence-informed public health decision making in Canada.

Numerous barriers to evidence-informed decision making and the information needs of decision makers have been identified [5,6]. Barriers include: difficulty keeping current with the large volume of evidence available; lack of access to full texts of journal articles; difficulty locating information specific and relevant to public health; difficulty accessing available evidence given the variations in search terms across databases; and difficulty tracking and saving search strategies and results [6]. User needs include: a one-stop-shop for evidence; automatic notification of updates regarding newly available evidence; and high quality synthesized evidence [6].

The best ways to attain an evidence-informed public health system in Canada have not been identified, despite numerous studies on the effectiveness of KTE strategies. The evidence suggests that KTE strategies need to be interactive [7-9] and involve face-to-face interaction [10-12]. Involvement of decision-makers in the research process has also been associated with higher degrees of uptake [13,14], and when the results or 'actionable messages' of research results are tailored to the specific needs of decision-makers then reported uptake is higher [1,4,7,15,16].

Knowledge management (KM) is emerging as a key factor contributing to the reduction of barriers to evidence-informed public health decision making [17-19]. KM is the systematic processes and resources used by individuals and organizations to identify, capture, store, retrieve, share, adapt, and (re)use tacit and explicit knowledge produced and/or needed by an organization [20,21]. A good KM strategy provides a multitude of functions that respond directly to the needs of its intended users [22]; is transparent; and adheres to a rigorous process. Technology, in particular Internet-based technology, has not only emerged in the past decade as a key tool in realizing KM but also as a popular strategy to promote evidence-informed decision making [5,23]. Certainly the potential of the Internet to reach large numbers of decision makers in a timely and cost-efficient manner is high, but whether Internet-enabled tools can facilitate evidence-informed decision making has yet to be determined.

One Canadian study evaluating the impact of the Internet as a KTE activity found that the dissemination of best practices information via the Internet to public health professionals encouraged participants to access information at other online sites compared to print-based dissemination [24]. Participants in this same study cited easy access to relevant information as a major benefit, saving managers from having to identify, access, and retrieve their own literature. When traveling to face-to-face meetings is not possible (as is the case for many front line public health professionals due to the current economic status), the Internet provides a valuable communication and networking opportunity [25], while facilitating research collaboration [26].

The purpose of this paper is to describe http://www.health-evidence.ca a knowledge management tool, which is one component of a more comprehensive knowledge management strategy being developed to facilitate evidence-informed public health decision making in Canada.

### Health-evidence.ca: A Knowledge Management Tool for Public Health

Systematic reviews and meta-analyses can be particularly powerful tools to inform and influence public health policy and practice decisions [27-29]. Furthermore, synthesized evidence provides a more consistent and conservative estimate of effect in comparison to individual studies [30-32]. Health Evidence is an organization whose mandate is to facilitate access to all systematic reviews and meta-analyses published since 1985 evaluating public health and health promotion interventions, as well as contribute to the development of capacity and culture for evidence-informed decision making. The website, http://www.health-evidence.ca is a key component of this developing KM system in Canada for front line public health professionals, policy-makers, research funders, researchers and students.

Work on the site began in April 2001 and the site was officially launched on March 10, 2005. A number of funded studies informed its development [12,33,34] and identified key functions and components preferred by public health decision makers. Results from these studies demonstrated a strong desire among Canadian public health decision makers for a national repository of research evidence, assessed for methodological quality and which could be accessed easily online [12,15]. Lapelle et al [6] reported similar findings among American public health decision makers in the year following the launch of health-evidence.ca.

When health-evidence.ca was launched, the short term (1-3 years) objectives were to: a) provide an easily-accessible source of published, reliable, up-to-date reviews evaluating the effectiveness of public health and health promotion interventions; b) act as a communication tool to facilitate exchange among Canadian public health and health promotion decision makers and researchers; c) build familiarity with the interpretation and integration of research evidence into the decision-making process; d) provide decision makers with tools to enhance their critical appraisal skills; e) customize the content received by decision makers to their specified areas of interest; and f) improve strategic networking and partnership building among researchers, decision makers, and practitioners, by providing opportunities for interaction. The long term objectives (5-10 years) included: a) being the go-to source for published reviews of public health and health promotion effectiveness; b) to host various online communities of practice (Canadian and international in scope) to promote knowledge translation and exchange; and c) to provide a mechanism for evaluating innovative KTE strategies.

Embedded functions of health-evidence.ca include:

• a user registration process that allows users to tailor the information they receive to particular areas of interest;

• a free-text search system that enables the use of commonly-used public health and health promotion terms;

• an assessment of the methodological quality of each review in health-evidence.ca;

• a sorting system that allows users to narrow search results by review quality (strong, moderate, or weak), topic area, intervention location, or type;

• a standardized short summary template (2-4 pages) for each review. Each summary frames the issue within a Canadian context and provides implications for policy and practice corresponding to each evidence point;

• a built-in feedback mechanism provides users with the opportunity to give suggestions for ongoing site improvement.

In addition to national consultation with public health decision makers, a search was conducted to determine if an evidence resource like http://www.health-evidence.ca already existed. Two organizations at that time had developed evidence resources that were particularly noteworthy. The Centre for Reviews and Dissemination had developed a number of databases including the Database of Abstracts of Reviews of Effects (DARE), the Economic Evaluation Database, and a database of Health Technology Assessments. The Evidence for Policy and Practice Information and Coordinating Centre (EPPI Centre) also had developed a database of reviews covering a broad list of topic areas. However, closer examination determined that these databases did not address a number of challenges experienced by public health decision makers. For example, public health decision makers wanted a database specifically focused on public health services and interventions and furthermore a database that housed reviews that evaluated public health interventions. While the above databases included reviews evaluating public health interventions, a significant portion of the sites' content was not public health related. This increased the likelihood that users of the site would find evidence not directly applicable to public health. Given that finding public health relevant evidence was a key challenge identified by public health decision makers in North America, this supported, at least in part, the need for developing a public health specific resource.

A second challenge identified by public health decision makers at the time was a lack of skill in critically appraising reviews and the desire to have a credible resource conduct the appraisal for them. Exploration of the databases above illustrated that DARE appraises methodological quality of systematic reviews in the database, and makes this assessment available to users of the site. However, an explicit assessment of the methodological quality of the reviews housed in the EPPI Centre database was not provided.

Finally, a key challenge expressed by public health decision makers in finding reviews of effectiveness was in identifying the correct keywords and MeSH terms to include as part of a search strategy. Assessment of the available databases in 2000 demonstrated a need for an evidence resource that provided an easy to use search alternative - one that did not require the use of MeSH terms and preferably provided a drop-down list of known terms to choose from. Since the available evidence resources in 2000 did not address a number of the key challenges and barriers identified by public health decision makers, the development of http://www.health-evidence.ca seemed necessary and warranted, if movement toward EIDM was to be achieved at some point in the future, at least in Canada.

## Methods

### Development Phase (April 2000 to March 2005)

**Figure 1:**
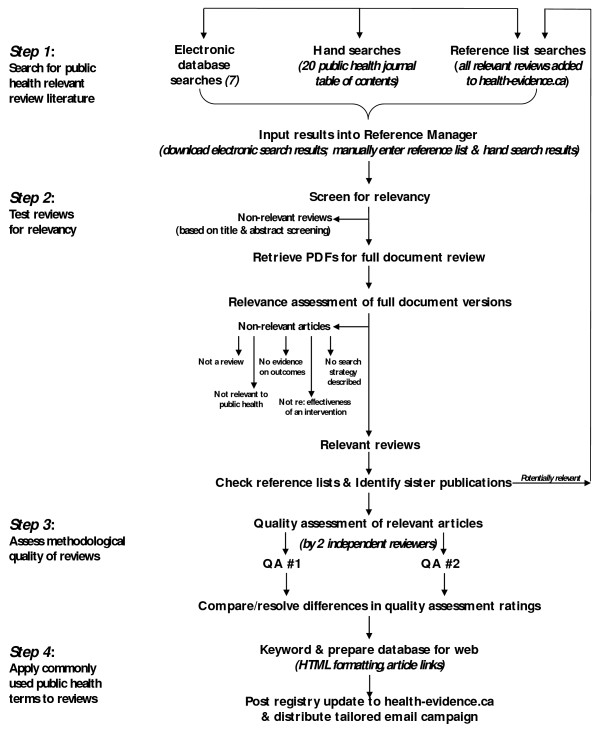
**Flowchart of Registry Process**.

Each review included in health-evidence.ca is subjected to rigorous processing prior to being posted on the site. This process is depicted in Figure 1. First we conduct an extensive search for reviews evaluating public health and health promotion interventions. Then we formally assess each review for relevance, assign a set of public health and health promotion keywords, assess the review for methodological quality, write a short summary of the review including implications for policy and practice, and finally post the review to the site. Links to the full text are provided so that users may access the full review where it is publicly-accessible online, or through IP authentication if users have existing journal or database (e.g., EBSCO/OVID) subscriptions.

The systematic reviews that populate health evidence.ca are identified through an extensive search (1985-present) that includes electronic searches of 7 databases: MEDLINE, EMBASE, CINAHL, PsycINFO, Sociological Abstracts, BIOSIS, and SportDiscus. For MEDLINE, EMBASE, and PsycINFO, the systematic review hedge used in PUBMED, which includes the following terms: systematic review.tw., meta-analysis.pt., and intervention$.ti (Medline version) or/1-4, was used. The effectiveness of this hedge and others has been shown by others to significantly reduce the number of articles retrieved while maintaining a high level of sensitivity for finding articles that are relevant [35,36]. We modified the hedge strategy slightly for EMBASE and CINAHL. When we compared the results of searches using the systematic review hedge to results of searches using the more general public health strategy, the systematic review hedge outperformed the latter [Lee, E., DeCorby, K., Dobbins, M.: Searching for reviews in the public health literature, unpublished]. For example, the systematic review hedge yielded 13259 articles in Medline and captured 186 of the 207 relevant articles indexed (sensitivity 89.9), compared to the general public health search which resulted in 46619 articles, and 191 relevant articles (sensitivity 92.3). Both searches resulted in a high number of relevant articles being identified. However, there was an almost four-fold difference between the precision scores between the two searches (1.1 vs. 0.3), meaning that the systematic reviews hedge captured a higher number of relevant articles while keeping the number of non-relevant articles captured low. This reduction in articles retrieved significantly decreased the number of articles needed to read from 244.9 down to 71.4 articles, meaning that only 72 articles rather than 245 articles would need to be screened in order to locate 1 relevant review. As a result, a decision was made to use this systematic review hedge as the basis for the search strategy and has been used since 2008.

**Figure 2:**
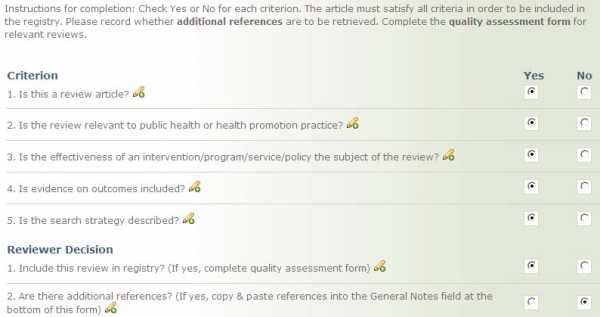
**Relevance Tool**.

In addition to electronic database searches, handsearching of more than 20 journals is conducted, and the reference lists of all relevant reviews are examined for additional references. Titles and abstracts (where publically available) generated from the searches are imported into Reference Manager and screened independently by two reviewers for relevance using a previously developed and tested tool (Figure 2). Reviewers receive training on the Relevance tool and pilot test the tool on a subset of reviews with ratings compared to those obtained by the first author (MD). Relevance criteria include: 1) is the article a review (must include the synthesis of more than one primary study); 2) is the intervention relevant to public health practice (a scan of public health departments and provincial governments identified the scope of public health practice in Canada); 3) is the effectiveness of an intervention evaluated; 4) is evidence on health outcomes reported; and 5) is the search strategy described (at least some description of how studies were identified must be provided). Reviews meeting all criteria are included in http://www.health-evidence.ca. Any discrepancies in ratings are resolved through consensus.

**Figure 3:**
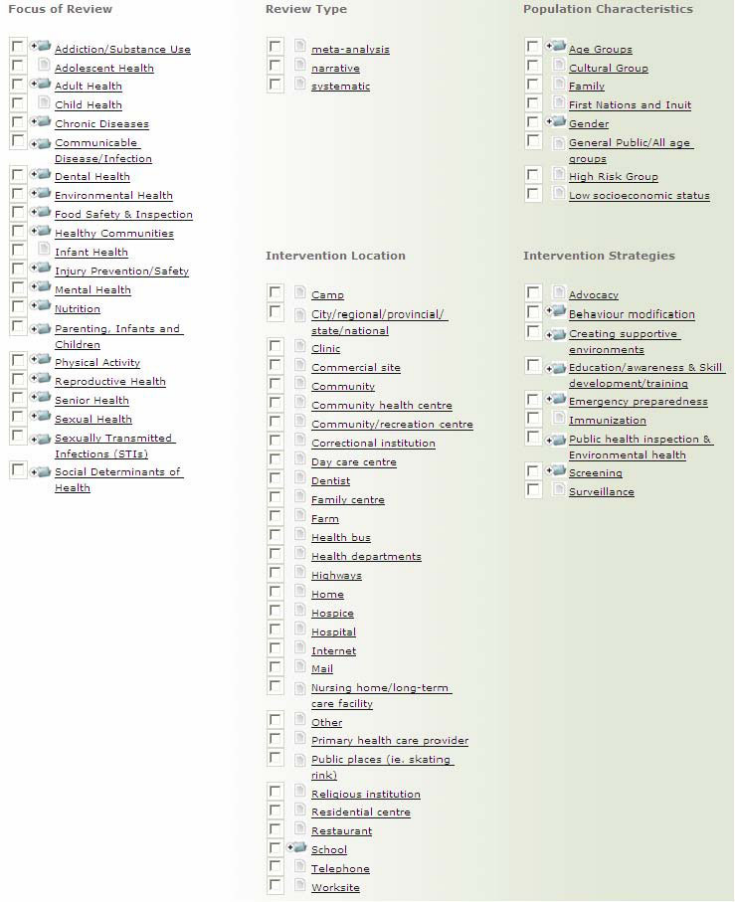
**Keywording Tool**. All keywords with a folder icon beside them are expandable and contain sub-keywords

Previous research has identified that keyword terminology (e.g. MeSH) employed in large databases, is difficult for public health decision makers to use, and represents a significant barrier to accessing research evidence [6]. To overcome this barrier, reviews on the site are assigned keywords using a tool developed by project staff through consultation with public health decision makers (Figure 3). Commonly used public health and health promotion terms are assigned to every review in the repository so as to facilitate ease of searching. Keyword terms are categorized into the following major themes: review focus, type of review, population or age group, intervention location, and intervention strategy. The keyword tool was tested on a sample of reviews independently by multiple project team members, until good agreement between reviewers was achieved. Keywording is completed by one staff member with any issues raised discussed in regularly scheduled team meetings, and periodic testing of the process by having two reviewers independently apply keywords on a sample of reviews and meeting to discuss any discrepancies.

**Figure 4:**
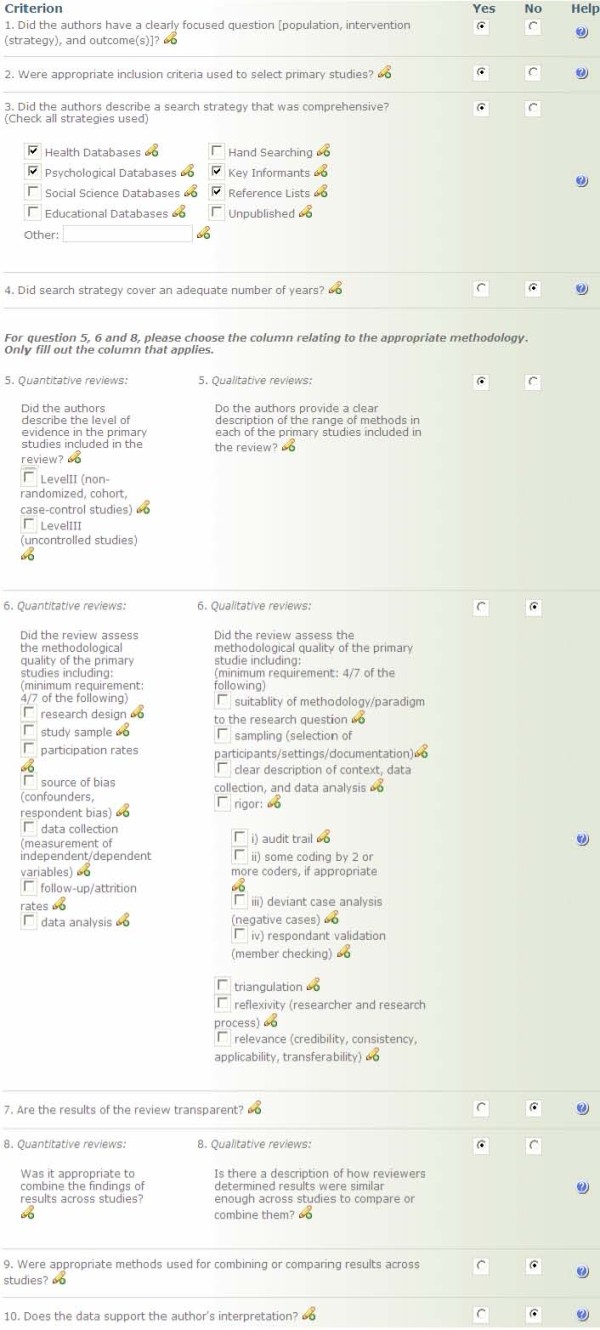
**Quality Assessment Tool**.

Relevant reviews are then assessed for methodological quality by two independent reviewers using a modified pre-existing tool [8] that has been assessed for reliability [37]. The tool is included in Figure 4. The ten criteria used to assess methodological quality are: 1) a clearly focused question was stated; 2) inclusion criteria were explicitly stated; 3) a comprehensive search strategy described; 4) adequate number of years covered in the search; 5) description of level of evidence provided; 6) assessment of the methodological rigor of primary studies conducted and results described; 7) methodological quality of primary studies assessed by two reviewers and level of agreement between reviewers provided; 8) tests of homogeneity or assessment of similarity of results across studies conducted and reported; 9) appropriate weighting of primary studies conducted; and 10) author's interpretation of results were supported by the data. Each criterion, worth one point each, is given equal weight in the overall methodological assessment score. Reviews are given an overall score out of 10 and are classified into three categories: Strong, Moderate, and Weak. Reviews receiving an overall rating of seven or more are considered strong, those with a score of five or six, moderate, and those with four or less, weak. Discrepancies are resolved by discussion.

While there are many quality assessment tools for appraising systematic reviews [38], this one was chosen because it had been tested and found to have acceptable reliability [37], and included key criteria as recently suggested by Moher [39]. Hearty debate continues concerning the assignment of overall quality ratings (e.g. strong, moderate weak), with some strongly encouraging ratings not be provided but rather an assessment of each component of the process of conducting a systematic review. Proponents of this latter approach suggest decision makers should decide for themselves if a review merits use after being made aware of its strengths and weaknesses [40]. However, our experience with public health decision makers is that they lack the necessary skills to interpret quality assessments that provide a commentary [40] per criterion rather than an overall score; and in fact, that they want someone credible, whom they trust to indicate if a review is 'good enough' to use in practice [12]. In responding to this identified need an overall assessment rating is provided for each review on the site. However, in addressing the controversy related to an overall rating, the completed quality assessment tool for each review is also provided so that users can assess for themselves how each criterion has been scored.

**Figure 5:**
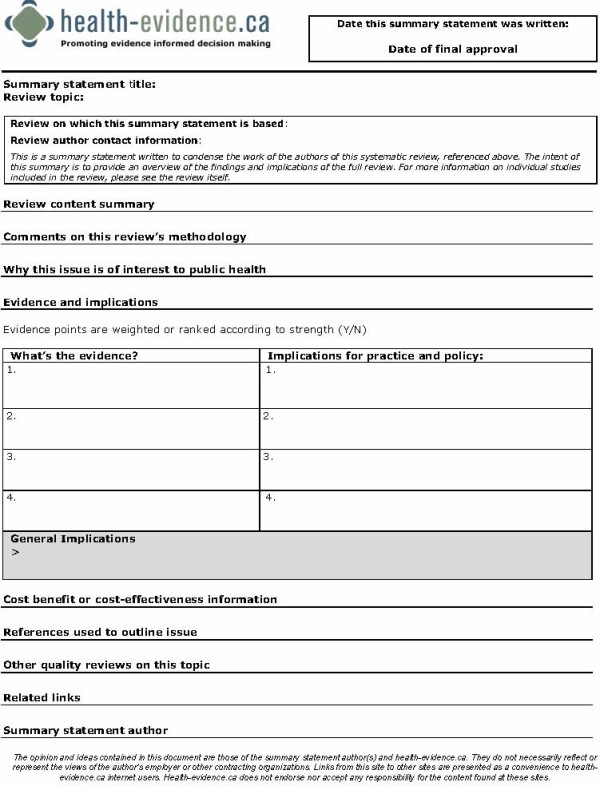
**Summary Statement Template**.

The next step in the process involves the writing of short, succinct summaries of the reviews. An example of the summary template is provided in Figure 5. The intent is that all reviews receiving a strong or moderate quality rating are summarized. The summary gives an overview of the review's content, the scope of the problem/issue within a Canadian context, the methodological quality of the review, and the key findings and implications for policy and practice. The summaries are written by health-evidence.ca staff. MD edits and approves the final version of all summaries before they are translated into French and then posted to the site. The goal of the summaries is to present the results of reviews in plain language, explicitly identifying the key action messages emerging from the review in a format that can be easily read and interpreted within two to three minutes. For example, an effect size provided in a meta-analysis with a confidence interval and/or level of statistical significance (p value) is explained in terms that could be interpreted by a reader with no statistical background. The language of the summary statements is consistent and easily transferable to decision makers at varying levels making the summary easy to use. A detailed guide on how to write a summary has been developed that clearly articulates what should be written for each section of the summary. The staff receives extensive training and feedback on summary writing. The process of writing a summary starts with research assistants writing Page 1 of the summary (summary of the review and its methodological quality and scope of the issue in Canada). Senior staff with experience in public health and health promotion (MD, PR, LG) write about the review results and implications for policy and practice, as well as the overall conclusions. Summaries are reviewed by at least two senior staff prior to final editing, translation and posting to the site.

### Maintenance Phase (March 2005 - Present)

Between March 2005 and the present, http://www.health-evidence.ca has been updated quarterly through ongoing electronic searches, reference list searches, and handsearches. Each quarter, approximately 9,500 titles and abstracts are screened and the set of potentially relevant reviews is reduced to approximately 200-250 references, which are retrieved in full text. Searches have been programmed in four of the electronic health databases (MEDLINE, EMBASE, CINAHL, and PsycINFO) to be executed automatically upon database update (every 1-2 weeks). Links to the automatically generated results are emailed to the project manager for importing and tracking of the results sets. The three remaining electronic health databases (BIOSIS, SportDiscus and Sociological Abstracts) continue to be searched annually. Handsearching is also done annually over the summer months, due to its human resource intensiveness, and accessibility of 4^th ^year undergraduate students or new graduates to assist in this activity. Students undergo extensive training in the process and all work is double rated by the project coordinator.

**Figure 6:**
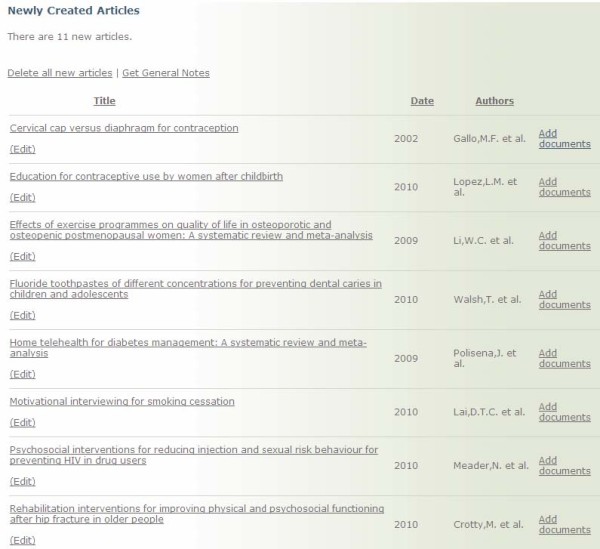
**Online Collaboration: New References, Ready for PDF Attachment**. PDFs attached via 'Add documents'

**Figure 7:**
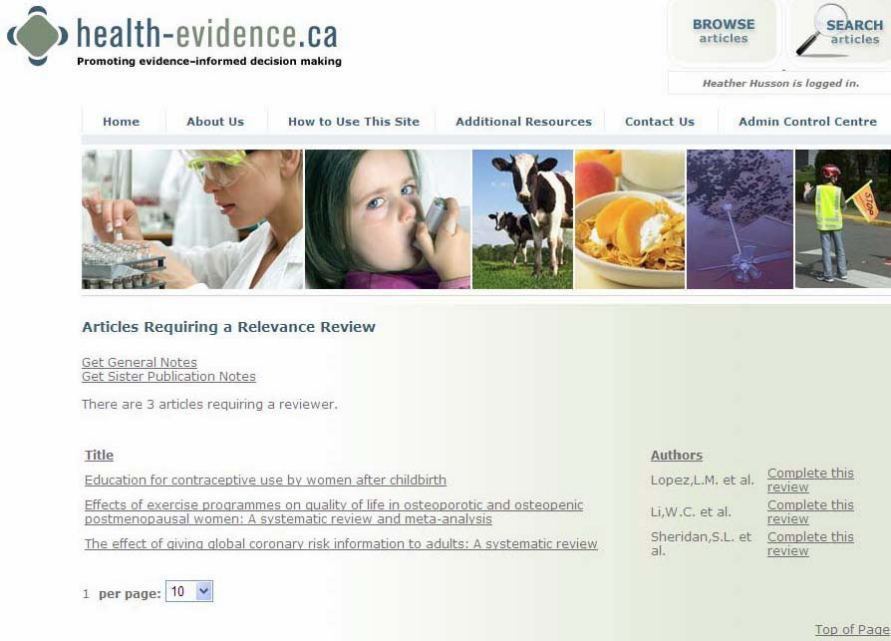
**Online Collaboration: Articles Requiring Relevance Queue**.

**Figure 8:**
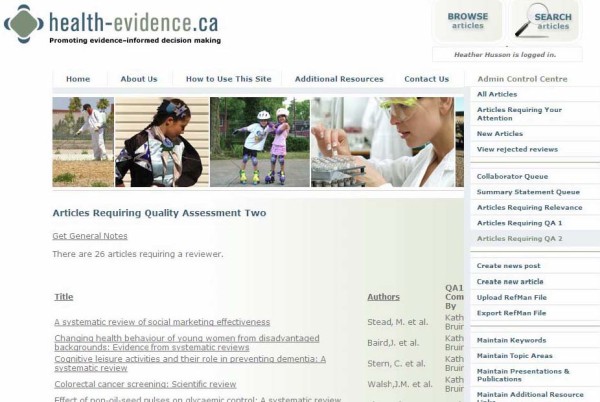
**Online Collaboration: Articles Requiring Quality Assessment Two Queue**.

Each quarter, potentially relevant references are uploaded to the website via XML files exported from Reference Manager. An online collaboration feature of the site allows for relevance testing, keywording, and quality assessment to be completed online. The online collaboration enables a completely paperless screening and assessment process, which is not publicly accessible. PDFs of reviews are uploaded and attached to each reference in the online system (Figure 6). Due to copyright restrictions, access to PDFs are only available to internal health-evidence.ca staff during the assessment process. Once a PDF is attached to a reference, the review moves to a queue for 'Articles Requiring Relevance' (Figure 7), where any designated reviewer is able to log on, select a review article from this queue, and begin relevance testing. Reviewers have the option to pass the review onto the quality assessment stage or reject the review (e.g. assess it as not relevant). Once a review has passed the relevance stage, it moves to the queue for 'Articles Requiring Quality Assessment 1', then onto 'Articles Requiring Quality Assessment 2' (Figure 8). Reviews are keyworded by the first reviewer at the Quality Assessment 1 stage, then the keywording is checked by the second reviewer at the Quality Assessment 2 stage. The system will not allow the same reviewer to complete both quality assessments, thereby ensuring each review is assessed independently by two reviewers. All reviews that receive the same quality assessment rating from each independent reviewer move to a holding queue where the project manager conducts a final check for completeness, then posts the article to the live site. Articles receiving different quality assessment ratings from the two reviewers are flagged for re-evaluation. Each reviewer receives an e-mail from the system notifying them that an article they reviewed needs to be reassessed. The two independent reviewers then log on and view their quality assessments in a side-by-side view to compare the discrepancies between reviewer ratings. Discrepancies are resolved through consensus.

The functionality of http://www.health-evidence.ca continues to be upgraded in response to user feedback, emerging evidence of effective knowledge translation strategies, and changing technology. One such upgrade involved updating the keywording tool. Public health services, in Canada and elsewhere, continue to evolve. As a result, the keywording tool requires periodic updating. Between 2005 and 2006 feedback on the keywording tool from site users, librarians, and others was accumulated. In 2006 we identified a handful of terms that had to be added to the keywording tool, including Social Determinants of Health, Men's Health, and Reproductive Health. Once a final list of new terms was decided upon and checked for correctness and acceptability, all reviews in http://www.health-evidence.ca at that time (approximately 1000) were re-keyworded and uploaded to the live site. This time-intensive activity was conducted by two staff members under the supervision of the project manager and took approximately two months to complete. The currency of keywords is important and represents a significant challenge to evidence resources like http://www.health-evidence.ca. While it is important for the keywords to adequately reflect the scope of public health practice, this must be paired with the human resource costs of completing this work. Furthermore, if the new keyword term is one that requires new database searches to be conducted, this has considerable resource implications. Fortunately, new database searches were not required during the keyword tool upgrade in 2006. However, if new database searches had been required, additional funding sources to support this activity would need to have been acquired.

A controlled vocabulary has also been added as a key function to the site. Feedback from users illustrated that decision makers with different educational or philosophical backgrounds use different terminology for the same phenomenon. We often are asked to change a keyword or add a new one. Given the resources required to re-keyword, we have developed a controlled vocabulary system that allows these additional terms to be integrated into one's search. For example, a user may have typed in the word breastfeeding in the free text search box. When the results are displayed a separate box will also appear asking the user if they would like to include a list of similar terms in their search; for example, a search for breastfeeding will also ask the user if they would like to search for lactation, infant nutrition, infant feeding and breast milk.

Additional tips on searching have also been built into the system. For example, if a search produced a large number of results or very few results, a message pops up stating *"Having trouble with your search?. Click here to view some useful tips on searching". *The 'tips' page makes suggestions on what to do if too many or too few results were found, as well as an additional section if more help is wanted. If users click on the link for more help, we suggest sending us their search question via the feedback loop. Staff conduct the search, and respond within one business day not only with the results but suggestions on the optimal combination of search terms.

Other improvements made to the site since its launch include: the ability to save in one's user file up to 50 search strategies; the ability to use AND, OR, NOT, for combining search terms; additional information on the search results page allowing users to see at a glance the title, author, year of publication and quality assessment rating for each review found; and a pop-up glossary function that can be turned on/off using a toggle switch located on the top menu bar. When enabled, common terms used throughout the site appear underlined and will have a definition pop up when scrolled over. This includes terms such as evidence-informed decision making, confidence interval, odds ratio, and relative risk, which help users optimally understand the site content.

## Results

### Health-evidence.ca: Content

To date more than 950,000 titles have been screened. As of June 21, 2010, 1913 reviews are posted on the site. Table 1 illustrates the number of reviews for each of the 21 main topic areas overall as well as by quality assessment rating. Because many reviews cover multiple topic areas the overall total in Table 1 is greater than 1913. Just over 78% of reviews have been assessed as being of strong or moderate methodological quality, with approximately 55% being rated as strong.

**Table 1:** Numbers of Review by Topic Area

Focus of Review	Methodological Rating			
	
	Strong	Moderate	Weak	Total
Chronic Diseases (All)	*329*	*144*	*141*	**614**

Adult Health (All)	*244*	*117*	*98*	**459**

Child Health	*208*	*86*	*75*	**369**

Nutrition (All)	*187*	*96*	*83*	**366**

Addiction/Substance Use (All)	*152*	*79*	*81*	**312**

Physical Activity (All)	*148*	*79*	*78*	**305**

Adolescent Health	*139*	*71*	*80*	**290**

Mental Health (All)	*156*	*75*	*53*	**284**

Parenting, Infants and Children (All)	*152*	*67*	*52*	**271**

Injury Prevention/Safety (All)	*166*	*57*	*43*	**266**

Communicable Disease/Infection (All)	*134*	*56*	*51*	**241**

Reproductive Health (All)	*131*	*43*	*40*	**214**

Sexually Transmitted Infections (All)	*81*	*48*	*58*	**187**

Sexual Health (All)	*87*	*46*	*38*	**171**

Infant Health	*81*	*32*	*23*	**136**

Senior Health (All)	*84*	*27*	*22*	**133**

Healthy Communities (All)	*43*	*26*	*29*	**98**

Environmental Health (All)	*39*	*10*	*14*	**63**

Social Determinants of Health (All)	*30*	*13*	*8*	**51**

Dental Health (All)	*28*	*9*	*10*	**47**

Food Safety & Inspection (All)	*8*	*2*	*2*	**12**

**Figure 9:**
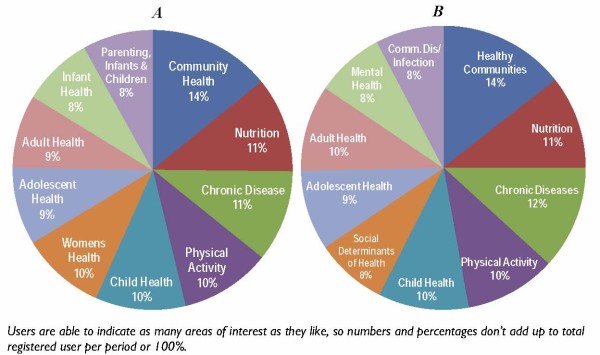
**Top 10 Areas of Interest Selected by Registered Users, December 15, 2005 & December 15, 2009**. A: December 2005 (n = 1510). B: December 2009 (n = 4206)

While users of the site are encouraged to register, it is not required to access any materials on the site. Registration however, enables us to tailor future communication to users' areas of interest. Site usage is monitored in two ways: usage by registered users and usage by all visitors to the site through Google Analytics. Since its launch, health-evidence.ca has attracted more than 4500 registered users from multiple countries, backgrounds, and interests. Approximately 80% of registered users are Canadian covering all provinces and territories, with remaining users from the United States, Australia and the UK. From registration data collected between March 2005 and January 2008 we know the largest user groups were public health nurses, program managers, health promotion workers, researchers, and program coordinators. We also know that the majority of users (67%) access the website from direct traffic (e.g. have the site bookmarked, or type it directly into their browser), 20% link to health-evidence.ca from a referring site, and 12.5% of users link from a search engine. While the topic interests of registered users have changed somewhat, generally interests have remained relatively stable since the site's launch, as is depicted in Figure 9.

Site usage has remained relatively stable in the past five years as illustrated in Table 2. Particularly noteworthy between 2005 and 2009 is the 25% increase in unique site visitors, the considerable increase in average time spent on the site per visit from 35 seconds to 4 minutes, and an 11% increase in Canadian users.

**Table 2:** Site Usage Statistics 2005 versus 2009

Website Visits Overview	*March 10, 2005 - December 14, 2005*	*March 10, 2009 - December 14, 2009*
**Total Unique Visitors**	12,780	17,111

**Total Site Visits**	31,314	27,640

**Average visits per day**	111	99

**Average visit duration**	0:35 seconds	4:03 minutes

**Visits from Canada**	52.1%	63.7%

Feedback from users has generally been positive. We are aware of the site being referenced in peer reviewed journals as either highlighting the site as a reference for public health decision makers [6,41-44] or identifying various tools available on the site of interest to public health decision makers [45-47]. Furthermore, the site was recently recommended for use in the Institute of Health Economics' annual report [48].

Recent efforts to move toward more active rather than passive knowledge translation strategies have been implemented. For example newsletters are disseminated quarterly to both individual users, as well as those who belong to other networks with whom health-evidence.ca is partnered. Electronic dissemination of the newsletter coincides with completion of the quarterly updates of new content being added to the site. This electronic newsletter summarizes the new content posted to health-evidence.ca, as well as categorizes links to reviews assessed as being of strong or moderate methodological quality according to the 21 main topic areas. This facilitates easier access to relevant evidence for any given user. More recently, tailored messages have been implemented whereby registered users receive emails with links only to those new reviews that relate to topic areas identified in their user profiles. Other active knowledge translation strategies recently implemented include a webcast which saw more the 200 registrants, a Fireside chat (online webinar), and video clips of User stories (users of health-evidence were interviewed and video clips of these interviews are posted on the site).

Monitoring of usage statistics over the past year indicates that important increases in site visits occur immediately following the release of the electronic newsletter and tailored messages. For example, the release of the March 2008 newsletter resulted in more than 500 site visits daily in the two weeks following its release in comparison to the annual average daily site visit rate of 100. These user statistics identify health-evidence.ca as an established key resource for public health and health promotion decision makers in Canada. While these statistics do not allow inference concerning the incorporation of this evidence into public health policy and practice decisions, the fact that many users return to the site multiple times each year suggests that this knowledge management tool is fulfilling a need identified among this target population. In addition, it is also worth noting that the most highly-accessed reviews coincide with some of the most pressing policy and practice issues faced by public health decision makers from front-line practitioners to senior policy makers. Reviews related to chronic disease detection, prevention, and management, including obesity prevention, the promotion of healthy eating, diabetes prevention, and cancer screening interventions have been the most frequently downloaded. This coincides with priority public health topics identified by the Canadian Public Health Association [49].

Of course an evidence resource such as this is not without its challenges. While adherence to quarterly updates is an ambitious goal this means that at any given time the site is not completely current. At best, users can be confident that a very high percentage of reviews evaluating public health interventions published from 1985 to four months prior to accessing the site are present. While strategies to reduce this time lag are continually ongoing (e.g. the online collaboration system reduced the time lag from six months to four months), four months is the best attainable given current resource levels. In addition, requests are received to expand the content of the site beyond public health. However, users of the site tell us that the value added of this site is that it includes only public health evidence. A significant challenge we face however is the constant change and structure of public health services. For example, emerging areas in public health include the built environment, climate control, and geospatial analysis. As new areas of public health emerge, so must the scope of the searches employed by http://www.health-evidence.ca. However, funding for such activities may be challenging to obtain and it is not entirely clear at this time who would be best suited to fund such activities.

Another significant challenge for health-evidence, as we reflect on the past five years and contemplate the next five years, is technological advances. In 2005 when http://www.health-evidence.ca was launched, the system was built using state of the art technology at the time. However, within two years this technology was already outdated. Significant investment in the site in 2007/2008 led to the introduction of many new features and functions that were enabled by moving to emerging technology. However, in 2010, we are faced yet again with significant improvements in technology (Web 2.0) and failure to keep up with technology will hamper our efforts to interact actively with an audience that is constantly becoming more computer savvy. However, to remain current with technology requires resources that are often not available in publically funded environments.

Finally, long term stable funding for http://www.health-evidence.ca and similar evidence resources represents a significant challenge and cause for concern. Currently in Canada there exists limited opportunities to obtain infrastructure funding to sustain such resources, despite evidence of use among target users. Efforts to develop sustainable funding partnerships in the long term are ongoing so as to ensure this resource and other similar resources in different disciplines remain freely accessible. However, long term funding is an issue that should be addressed prior to the creation of similar such evidence resources in the future.

### Future Plans for Site Development

Future goals for health-evidence.ca are to catalogue and make available the full text of all published review evidence on the effectiveness of public health interventions and to provide summaries of that evidence in both English and French. While health-evidence.ca provides all static content pages such as Additional Resources, site information, and tools, in French as well as English, funding has not yet been available to translate all of the reference material on the site, nor to screen, appraise, and summarize any French-language reviews encountered in the electronic database searching. However, efforts are ongoing to secure the resources to conduct this work. Health-evidence.ca is also prioritizing the translation of summaries for well-done (strong or moderate quality) reviews housed in the registry. Upcoming improvements to health-evidence.ca include expansion of the static content on the site, particularly related to evidence-informed decision making resources, and the exploration of search strategies for the published literature in zoonoses and causation questions for public health decision making. Research proposals have been submitted to national and provincial funding bodies to systematically and rigorously evaluate the impact of the site on evidence-informed public health decision making.

## Conclusion

Knowledge management has recently been recognized as necessary for the delivery of effective public health services, and as a significant factor in the realization of evidence-informed public health decision making. This paper describes one example of a knowledge management tool for public health in Canada. It is encouraging to see the level of site usage by public health decision makers in Canada and abroad, as well as the accessing of reviews that correspond with current public health priorities. While this does not imply that http://www.health-evidence.ca is influencing public health policy and practice, it does illustrate awareness of this resource by the intended audience, which is a first step in the evidence-informed decision making process. However, it is important that knowledge management tools such as these be evaluated rigorously to determine not only if they are being accessed and used, but that they impact policy and program planning. Ongoing efforts to evaluate the site are underway and will be reported upon as results become available.

## Competing interests

The authors declare that they have no competing interests.

## Authors' contributions

MD participated in the design of research studies related to the site, wrote the initial draft of the manuscript and incorporated contributing author feedback into the paper. PR contributed to all drafts of the manuscript. KD contributed to all drafts of the manuscript. DT collated statistics for the site. HH provided site feedback. LG provided feedback on the draft. All authors read and approved the final manuscript.

## Pre-publication history

The pre-publication history for this paper can be accessed here:

http://www.biomedcentral.com/1471-2458/10/496/prepub
